# Social, Genetics and Histopathological Factors Related to *Titin* (*TTN*) Gene Mutation and Survival in Women with Ovarian Serous Cystadenocarcinoma: Bioinformatics Analysis

**DOI:** 10.3390/genes14051092

**Published:** 2023-05-16

**Authors:** Fabiana de Campos Gomes, Eric Renato Lima Figueiredo, Ediane Nunes De Araújo, Edila Monteiro De Andrade, Carlos Diego Lisbôa Carneiro, Gabriel Mácola De Almeida, Helana Augusta Andrade Leal Dias, Lucélia Inoue Bispo Teixeira, Manuela Trindade Almeida, Mariusa Fernandes De Farias, Natália Albim Linhares, Natasha Lima Da Fonseca, Yago Dos Santos Pereira, João Simão de Melo-Neto

**Affiliations:** 1 Postgraduate Program in Health, Environment and Society in the Amazon (PPGSAS), Federal University of Pará (UFPA), Street Augusto Corrêa, 01, University City: José Silveira Neto, Health sector: Guamá, Belém 66075-110, PA, Brazil; 2 Faculty of Medicine CERES (FACERES), São José do Rio Preto 15090-305, SP, Brazil

**Keywords:** ovarian serous cystadenocarcinoma, titin gene (*TTN*) mutation, bioinformatics analysis, genetic factors, survival

## Abstract

Several factors may increase the risk of development of ovarian cancer. In this study, we investigated the relationship between social, genetic, and histopathologic factors in women with ovarian serous cystadenocarcinoma and titin (*TTN*) mutations, whether the *TTN* gene mutation may be a predictor, and its impact on mortality and survival in these patients. A total of 585 samples from patients with ovarian serous cystadenocarcinoma were collected from The Cancer Genome Atlas and PanCancer Atlas through the cBioPortal for analysis of social, genetic, and histopathological factors. Logistic regression was used to investigate whether *TTN* mutation could be a predictor, and the Kaplan–Meier method was applied to analyze survival time. *TTN* mutation frequency did not differ between age at diagnosis, tumor stage, and race, and was related to increased Buffa hypoxia score (*p* = 0.004), mutation count (*p* < 0.0001), Winter hypoxia Score (*p* = 0.030), nonsynonymous tumor mutation burden (TMB) (*p* < 0.0001), and reduced microsatellite instability sensor score (*p* = 0.010). The number of mutations (*p* < 0.0001) and winter hypoxia score (*p* = 0.008) were positively associated with *TTN* mutations, and nonsynonymous TMB (*p* < 0.0001) proved to be a predictor. Mutated *TTN* affects the score of genetic variables involved in cancer cell metabolism in ovarian cystadenocarcinoma.

## 1. Introduction

Ovarian cancer is the sixth most common and deadliest malignancy in women [1]. The incidence rate varies among different countries and is directly related to demographic and social factors [2]. A high incidence has been reported in women close to 60 years of age, but individuals with a family history of ovarian cancer may develop the disease approximately 10 years earlier [1].

Regarding mortality, a study based on the Global Cancer Observatory (GLOBOCAN) database showed that mortality rates vary among different countries due to differences in the Human Development Index (HDI) [2]. According to the National Cancer Institute (INCA), in Brazil, ovarian cancer is considered a gynecologic neoplasm with the lowest chance of cure, because of the challenges in diagnosis and the lack of an appropriate screening method to detect the disease at an early stage [3]. In this context, the discovery of a biomarker considering the histological subtypes of ovarian cancer may help identify individuals at a higher risk and/or detect the disease at an early stage.

Histologically, ovarian carcinoma is divided into five types: serous, endometrioid, mucinous, clear cells, and squamous cells, which are further divided in subtypes [4,5]. Among the serous types, ovarian serous cystadenocarcinoma (OSC) is the most common epithelial histological subtype. However, due to lack of screening methods, there are limitations in detecting the disease at an early stage, which contributes to difficulties in treatment and cure [6]. This may lead to disease progression, which in turn has an impact on mortality rates. Therefore, screening methods, e.g., using molecular biomarkers, are of great importance for the diagnosis and identification of risk factors, such as genetic, demographic, and social factors associated with women who are more susceptible to the disease.

Regarding genetic factors, although certain gene mutations, such as breast cancer gene 1 and 2 (*BRCA1/2*), tumor protein p53 (*TP53*), epidermal growth factor receptor (EGFR), and AKT serine/threonine kinase 2 (*AKT-2*) [7], are involved, other mutated genes may also affect the development and progression of ovarian cancer subtypes [8].

A recent study showed that a spontaneous mutation of the titin gene (*TTN*) represents a tumor mutation burden (TMB) associated with various cancers, including ovarian cancer [9]. The *TTN* gene acts in chromosome condensation and segregation (https://genome-euro.ucsc.edu/ accessed on 23 December 2022). Therefore, the presence of a mutation can compromise gene function at this stage of the cell cycle and favor the emergence of neoplasms, such as ovarian cancer. Analysis of the frequency of this mutation in the Catalogue Of Somatic Mutations In Cancer (COSMIC) database (http://www.sanger.ac.uk/cosmic accessed on 23 December 2022) [10] revealed that this gene has the highest mutation frequency in this histological type. However, TTN mutation in ovarian cancer, specifically in OSC, and its relationship with social, clinical, and genetic factors have not been studied, although it is clinically relevant and fundamental to determine its applicability in clinical practice. An investigation into the relationship between *TTN* mutation and OSC may enable the discovery of a new biomarker for the screening and management of the disease in clinical practice.

In addition, analysis of mutation types, the presence of microsatellite instability (MSI), and *TTN*-related hypoxia scores can provide information regarding important characteristics of the tumor and establish relationships with tumor severity and progression in affected individuals [11,12]. Therefore, the evaluation of these parameters in relation to *TTN* mutation may provide evidence for a new prognostic and predictive biomarker for the risk and aggressiveness of ovarian cancer in women with the mutated gene.

In this study, an analysis of OSC samples from The Cancer Genome Atlas (TCGA) was performed to identify the relationship of *TTN* gene mutation with social, genetic, and histopathological factors in women diagnosed with OSC, to verify whether this mutation can be a predictor and if it impacts the mortality and survival of these individuals.

We hypothesized that social, genetic, and histopathological variables are related to the presence of *TTN* mutations. Furthermore, it is hypothesized that *TTN* mutation may be related to a higher risk of OSC, increased mortality, and reduced survival in women diagnosed with OSC.

Thus, the results of this study may contribute to early disease screening and the development of personalized treatments for patients with OSC and provide a better understanding of the molecular mechanisms of the disease.

## 2. Methods

### 2.1. Ethical Aspects

The open-access database used in this research is maintained by the Memorial Sloan Kettering Cancer Center (MSK). These individuals were not identified and contained anonymous data; therefore, according to the National Health Council (CNS) Resolution No. 510, 7 April 2016, evaluation by the research ethics committee was not required [13].

### 2.2. Study Design

Observational study with ecological design having descriptive and inferential bioinformatics analysis.

### 2.3. Population and Period of Study

The study included 585 women diagnosed with OSC and the relevant data were collected in a public database from TCGA, PanCancer Atlas through the cBioPortal [14]. Secondary data were accessed and extracted from public databases in October 2022 and constituted the main source of analysis in this study.

### 2.4. Inclusion and Exclusion Criteria

As inclusion criteria, we selected genomic samples with OSC, ICD-10: C56.9 (malignant ovarian neoplasm), C48.2 (malignant neoplasm of the peritoneum), and C48.1 (malignant neoplasm of specific parts of the peritoneum), using the filter to select samples with “mutation” Molecular Profile for TTN gene mutation types (ENST00000591111.5 transcript). As exclusion criteria, samples with incomplete information were not considered for the analysis.

### 2.5. Data Base

Data were collected using the database publicly available on the cBioPortal platform, an open source and open access resource for the interactive exploration of multidimensional cancer genomics datasets (https://www.cbioportal.org accessed on 23 December 2022), maintained by the Center for Molecular Oncology at MSK [14].

The data access policy regarding the availability of data for publication was consulted on the MSK Data Catalog platform (https://datacatalog.mskcc.org/ accessed on 23 December 2022), which it defined as free for steps data OSC (TCGA, PanCancer Atlas) [15].

For the *TTN* gene, a prior consultation was performed in the OncoKB portal. OncoKB is a knowledge base platform in precision oncology, which was developed at the MSK and is composed of biological and clinical information regarding genomic alterations in cancer (https://www.oncokb.org/cancerGenes accessed on 23 December 2022) [16]. The consultation revealed that *TTN* has not been included in the list of cancer genes, highlighting the importance of studies assessing the potential of *TTN* as a cancer gene.

### 2.6. Data Extraction and Variables

Data on 1838 clinical samples of serous ovarian cancer were available on the platform. For this study, only data referring to OSC derived from TCGA were selected via the cBioPortal.

We used a dataset containing clinical and summarized data from a sample of 585 serous ovarian cystadenocarcinomas of 585 patients from the PanCancer Atlas initiative, which comprehensively addresses cancer issues. To obtain these samples, the filters Mutation (*n* = 475) and Genomic Profile for *TTN* mutation (*n* = 110) were used.

For analysis, we considered the following social variables: diagnosis age and race/skin color; genetic factors such as *TTN* mutation, mutation count, aneuploidy score, Buffa hypoxia score, fraction of genome altered, MSI MANTIS score, MSIsensor score, mutation count, Ragnum hypoxia score, Winter hypoxia score, nonsynonymous TMB, and histopathological information through grade histologic neoplasm. For survival analysis, we used data on disease-specific survival status (death present or absent), time of overall survival (months), and disease-free survival (months).

### 2.7. TTN Mutation Analysis

The frequency by mutation type for the TTN gene, specifically for the ENST00000591111 transcript in OSC samples, was checked using cBioPortal. To explore the hotspots of somatic mutations in the protein domains, the MutationAligner platform (http://www.mutationaligner.org accessed on 23 December 2022) was used [17].

### 2.8. Statistical Analysis

Descriptive analysis was expressed as frequency (absolute and relative), central tendency (median), and dispersion (interquartile range). To verify normality, the data were submitted to Kolmogorov–Smirnov test. To determine whether there were statistically significant differences between the frequency and median, the chi-square (χ^2^) or Mann–Whitney test was used.

Binary logistic regression analysis was used to determine whether the Titin (*TTN*) gene mutations could be a predictor. Initially, we performed univariate analysis considering a *p*-value < 0.20. To verify multicollinearity, the variance inflation factor (VIF) was calculated, and variables that presented a VIF value > 10 were removed from the final model. Statistical significance was set at *p* < 0.05. Odds ratios (OR) with 95% confidence intervals (95% CI) were used to quantify the degree of association.

For survival analysis, the Kaplan–Meier estimator was used to construct curves, and the log-rank (initial), Breslow (intermediary), and Tarone–Ware (final) tests were used to identify the occurrence of a statistically significant difference in different periods.

For statistical analyses, we used the Statistical Package for the Social Sciences (SPSS) Version 26.0 (IBM Corp. Released 2019. IBM SPSS Statistics for Windows, Version 26.0. IBM Corp., Armonk, NY, USA).

## 3. Results

### 3.1. Mutation of TTN and Histotype of Ovarian Cancer

In this study, a sample of 585 individuals (475 without the mutation for the *TTN* gene and 110 with the mutation) were analyzed to assess the impact of *TTN* mutation on social variables, grid histopathology, and genetic factors.

### 3.2. Mutation of TTN and the Relationship of Social, Clinical and Histopathological Factors

A sample of 585 individuals (475 without the mutation for the *TTN* gene and 110 with the mutation) were analyzed to assess the impact of *TTN* mutation on social variables, grid histopathology, and genetic factors.

Table 1 presents the social and clinical variables of women with ovarian cystadenocarcinoma with and without *TTN* gene mutation. The Buffa hypoxia score (*p* = 0.004), mutation count (*p* < 0.0001), Winter hypoxia score (*p* = 0.030), and nonsynonymous TMB (*p* < 0.0001) were higher in samples with mutation. Additionally, the MSI sensor score (*p* = 0.010) was lower in individuals with mutation.

As shown in Table 1, the analysis of both sets of data for the *TTN* gene showed that there was no significant statistical difference in relation to age at diagnosis (*p* = 0.419) and for race/skin color (*p* = 0.152), grade histologic neoplasm (*p* = 0.420), death (*p* = 0.937), and survival time (*p* = 0.082). However, one-tail analysis showed a lower survival time in the group with mutation (p = 0.041) (No *TTN* gene mutation: 34 (39) days; Mutation in *TTN* gene: 31 (35) days).

### 3.3. Analysis of Titin (TTN) Gene Mutation as Predictive to Social and Clinical Variables of Women with Ovarian Serous Cystadenocarcinoma

In the univariate analysis presented in Table 2, the Buffa hypoxia score (OR = 1.035 [95% CI: 1.012, 1.058], *p* = 0.003), MSI sensor score (OR = 1.206 [95% CI: 1.042, 1.396], *p* = 0.012), mutation count (OR = 1.010 [95% CI: 1.005, 1.014], *p* < 0.0001), and Winter hypoxia score (OR = 1.025 [95% CI: 1.007, 1.045], *p* = 0.008) were associated positively with *TTN* mutation. The other variables were not different in relation to *TTN* mutations.

In this sequence, multicollinearity of the variables was analyzed. The mutation count was removed for VIF values > 10. In the multivariate analysis, nonsynonymous TMB (aOR = 1.616 [95% CI: 1.346, 1.941], *p* < 0.0001) was found to be a predictor.

### 3.4. Survival Rates

**Figure 1:**
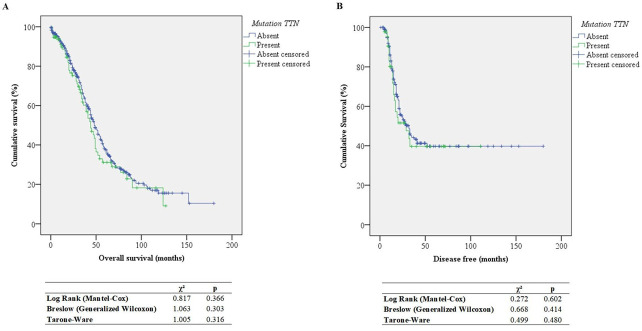
Kaplan-Meier curve for overall (**A**) or disease free (**B**) survival status in individuals with *TTN* mutation.

The Kaplan–Meier survival analysis is presented in Figure 1. There was no difference between overall or disease-free survival status with and without *TTN* gene mutations in different regions of the curve.

### 3.5. TTN Mutations in Ovarian Cancer

**Figure 2:**
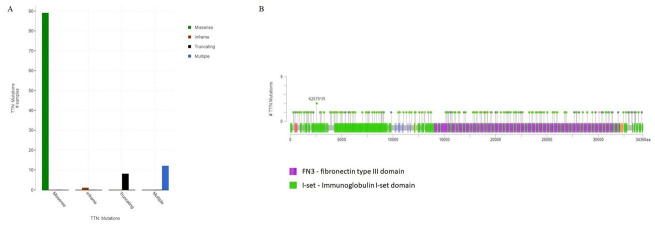
Mutation types for the ENST00000591111.5 transcript of the TTN gene. (**A**) Mutation types in samples from ovarian serous cystadenocarcinomas. (**B**) Hotspots of missense mutation indicating FN3 and I-set.

We evaluated the mutation types of the ENST00000591111.5 transcript of the *TTN* gene in OSC samples according to the data available in TCGA. Missense mutations were the main mutation type (89 samples), followed by in-frame mutations (1 sample), truncations (8 samples), and multiple mutations (12 samples) (Figure 2A). In terms of frequency by type of mutation, 181 mutations were identified at different sites of the gene, including missense (133), truncation (43), in-frame (1), and splicing (4) mutations (Figure 2B).

For missense mutations, the observed hotspots were fibronectin-type III (fn3) domain and the I-set immunoglobulin domain. Data from the InterPro description on the MultationAligner platform showed that fn3 domain has several important functions, including cell differentiation and migration, tumor metastasis, and cell adhesion activity. I-set domains are found in cell adhesion molecules, such as vascular, intercellular, and angiogenic molecules.

## 4. Discussion

OSC is the most common malignant ovarian tumor, accounting for approximately 50% of all cases. It occurs bilaterally in 30–50% of patients, affects women close to 60 years of age, is generally diagnosed late, and results in high lethality. In the context of late diagnosis and high lethality [9], studies on tumor biomarkers are very important for the prevention and early detection of ovarian cancer [3,6], as well as for analyzing social and demographic aspects to track the most vulnerable population and better understand the etiology of ovarian cancer [1,18,19].

In the present study, social factors were not found to be associated with *TTN* mutations. Social factors, such as age at diagnosis and race did not affect the frequency of *TTN* gene mutations. Previous studies have found that mutation in this gene is observed in other types of tumors, but not in the variables of social factors in this case, which represent trends between populations. [9,20]. To date, the link between *TTN* mutation and social, clinical, and genetic factors in ovarian cancer remains unexplored. Therefore, in this study, considering the database used and the Tissue Source Site, it is probable that the individuals are present in a certain geographical area that does not have large heterogeneity in social and demographic aspects [21].

The frequency of TTN_ENST00000591111 based on the type of mutation, primarily missense mutation, is relevant in serous carcinoma, indicating that it may be a promising marker for detecting the type of missense mutation related to *TTN* in ovarian cystadenocarcinoma. In this type of mutation, there is a variation in the DNA nucleotide in the coding region of the protein, which results in an alteration of the amino acid, and consequently of the codon, thereby influencing the structure, function, and level of the protein [22]. Therefore, there is a need for studies on genetic characteristics based on the histological types of ovarian cancer [23], as well as the identification of biomarker signatures to assess possible ovarian cancer [24]. The presence of *TTN* mutation in OSC may help characterize this histological subtype using a genetic marker.

Histopathological factors were not associated with *TTN* mutations. This is likely due to the fact that the *TTN* ENST00000591111.5 transcript is a key component of striated muscles, with low levels of reads per kilobase of transcription per million reads mapped in ovarian tissue (https://genome-euro.ucsc.edu/ accessed on 23 December 2022). This may explain the lack of a histological relationship with *TTN*.

Additionally, few studies have linked *TTN* mutations to cancer cell activity and prognosis of oncogenic mutations in ovarian cancer. An analysis of the genomic sequencing profile of primary epithelial ovarian cancer samples showed that somatic mutations in multiple genes including *TTN* can be a biomarker for early diagnosis [25].

Ovarian cancer is a complex and heterogeneous disease with multiple genetic and environmental factors contributing to its development. Several genes have been identified as important players in the onset and progression of ovarian cancer. *BRCA1/2* are two of the most well-known genes associated with the disease, and mutations in these genes increase the risk of developing both ovarian and breast cancer [26]. *TP53* is another commonly mutated gene in ovarian cancer, and its mutations are often associated with poor prognosis and resistance to chemotherapy [27]. Additionally, recent studies have identified mutations in the *AKT-2* gene as a potential contributor to ovarian cancer development and progression [28]. These genes are considered promising targets for screening and early detection as well as for developing new therapeutic strategies for ovarian cancer. For example, *BRCA1* and *BRCA2* genes have been successfully targeted with poly (ADP-ribose) polymerase (PARP) inhibitors, leading to significant improvements in response rates and overall survival in patients with *BRCA*-mutated ovarian cancers [29]. However, it has been observed that inhibitors of the DNA damage response (DDR) may be most active in cells that lose control of the G1/S checkpoint, which is related to the loss of function of TP53 [30]. Despite advances in the understanding of the role of these genes in ovarian cancer, more research is needed to thoroughly understand their contribution to the disease and to develop new and more effective screening and therapeutic strategies. In summary, *BRCA1/2, TP53*, and *AKT-2* are among the most relevant gene factors associated with ovarian cancer, and their study is critical for improving our understanding and management of this disease.

However, a recent study indicated that *TTN* is associated with high TMB [9]. Tumors with a high mutation burden have a greater capacity to generate neoantigens and become immunogenic, which directly influences the course and outcome of treatment [31]. In clinical practice, TMB is one of the Food and Drug Administration (FDA)-approved predictive biomarkers for immunotherapy [32,33]. Therefore, considering previous findings and the results of the present study, it is possible that the presence of a *TTN* mutation in OSC can also be used as a marker that should be further investigated in preclinical studies to test the in vitro activity of immunotherapy in a selected population with likely high TMB. However, the OncoKB portal showed no results regarding the *TTN* gene [16], emphasizing the importance of conducting this research.

Therefore, considering previous findings and the results of the present study, it is possible that the presence of a *TTN* mutation in OSC can also be used as a marker that should be further investigated in preclinical studies to test the in vitro activity of immunotherapy in a population with likely high TMB selected as a biomarker.

In this study, we found that genetic factors are associated with *TTN* gene mutation and OSC. The mutation count was higher in women with ovarian cystadenocarcinoma and was positively associated with *TTN* gene mutation. The *TTN* gene is susceptible to cancer protein kinase, encodes the major polypeptide expressed in oncogenesis, and is found in many functional cell types [34]. In particular, the *TTN* ENST00000591111.5 transcript plays an important role in chromosomal condensation and segregation during mitosis [35]. Therefore, mutations in the *TTN* gene may affect cell cycle and contribute to tumor occurrence and progression. In addition, we found that missense point mutations in the *TTN* transcript for the fn3 and I-set domains can affect cellular processes such as angiogenesis.

When we examined the relationship between Winter and Buffa hypoxia scores, we found an increase in hypoxia with *TTN* gene mutation. When a tumor develops, the homeostasis of tissue oxygenation is altered because of the increased demand for oxygen by the cancer tissue, leading to the rapid and erratic formation of new blood vessels and increased demand with regard to the available supply of oxygen [36]. This is known as the hypoxia score. Increased resistance to cancer treatment is just one of the many effects of hypoxia in cancer, which also has an impact on cell invasion and metastasis. In addition, survival is affected by hypoxia scores [37]. Although the analysis of the survival curve did not demonstrate significance, this study’s finding that the median survival time was lower in women with mutations supports the outcome. The findings of this research may help us better understand the tumoral environment because the association between *TTN* mutation and hypoxia score in ovarian cancer has not yet been researched.

Molecular analysis of mutation types, MSI status, and *TTN*-related hypoxia score indicated that dysregulation of these parameters can offer valuable information about tumor characteristics and its relationship with disease progression and severity in affected individuals [38]. Tumor biomarker testing is very important to detect the presence of underlying pathology derived from genomic alterations associated with the risk of ovarian cancer in family history. Additionally, recent studies have indicated a correlation between specific mutated genes and the histological subtypes of ovarian cancer [23], thereby highlighting the importance of incorporating molecular analysis into the overall understanding and management of ovarian cancer.

The MSI sensor score was lower in individuals with mutations but was positively correlated with a mutant *TTN* gene as the score increased. It is anticipated that MSI (a score that precisely evaluates microsatellite instabilities) will be increasingly employed in cancer diagnostics because it is crucial for understanding cancer progression, extracting information about familial risk, and developing treatment methods [39].

When women with ovarian cystadenocarcinoma had the *TTN* gene mutation, and the levels of nonsynonymous TMB were greater and more positively correlated. TMB is a biomarker for immuno-oncology that has a high rate of immunotherapy response and is used to quantify the burden of neoantigen tumors by examining both the quantity and quality of mutations [20,40]. Although tumor cells with high TMB tend to produce more immunogenic neoantigens, which are recognized by host T cells, especially cytotoxic T lymphocytes, and are among the most crucial factors in predicting chemotherapy response, TMB has been shown to be a promising predictor of mutations [41,42,43]. Our research corroborates the finding that *TTN* mutation serves as a risk factor for ovarian cancer [44].

Sociodemographic variables, such as family history, demography, and low/high income, were not analyzed because of database limitations. In addition, given the different natural histories and clinical backgrounds of the five ovarian cancer histotypes, the results of this study are only applicable to the analyzed subtypes. Nevertheless, our study limitation concerns the ecological design, where association between clusters does not necessarily indicate that it occurs at the individual level, when investigating samples from secondary databases [45]. However, the findings of our study can be valuable when hypothesizing future study designs, considering the possibility of using bioinformatics tools in the era of genomic cancer [46,47].

## 5. Conclusions

Social and histopathological factors were not associated with *TTN* mutations. However, we identified genetic factors associated with *TTN* mutations in women diagnosed with OSC. The Buffa hypoxia score, mutation count, Winter hypoxia score, and nonsynonymous TMB were higher and positively associated with *TTN* gene mutation in women with ovarian cystadenocarcinoma. MSI sensor scores were lower in those with mutation but were positively associated with mutated *TTN* gene. The survival time was lower in women with *TTN* mutation. Nonsynonymous TMB proved to be a predictor of interaction with other variables. In addition, missense point mutations in the *TTN* transcript for the fn3 and I-set domains may affect cellular processes such as angiogenesis. In summary, these findings indicate that *TTN* mutations and TMB may act as target genetic markers to assist in the detection of OSC and in the clinical management of the disease.

## Figures and Tables

**Table 1:** Social and clinical variables of women with ovarian cystadenocarcinoma with and without Tintin (*TTN*) gene mutation.

Variables	No *TTN* Gene Mutation (*n* = 475)	Mutation for *TTN* Gene (*n* = 110)	Total	U or χ^2^	*p* Value
**Age of Diagnosis**	57 (16)	58 (19)	57 (17)	17,445	0.419
**Race/skin color**				5282	0.152
American Indian or alaska native	1 (0.3)	2 (2.20)	3 (0.7)		
Asian	12 (3.3)	5 (5.4)	17 (3.7)		
Black or African american	23 (6.4)	7 (7.6)	30 (6.6)		
White	326 (90.1)	78 (84.8)	404 (89)		
**Genetic factors**					
Aneuploidy Score	13 (11)	12.5 (11)	13 (11)	21,694	0.274
Buffa Hypoxia Score	11 (21)	15 (17)	13 (18)	5694	0.004
Fraction Genome Altered	0.55 (0.22)	0.54 (0.25)	0.55 (0.23)	24,760	0.849
MSI MANTIS Score	0.28 (0.03)	0.29 (0.03)	0.28 (0.03)	17,691	0.985
MSI sensor Score	0.97 (0.85)	0.80 (1.15)	0.90 (0.89)	18,559	0.010
Mutation Count	68 (37)	83.5 (62)	69 (48)	10,564	<0.0001
Regnum Hypoxia Score	8 (10)	8 (8)	8 (10)	7002	0.448
Winter Hypoxia Score	10 (22)	12 (23)	12 (22)	61.39	0.030
TMB nonsynonymous	2.2 (1.3)	2.8 (2.1)	2.4 (1.6)	106.49	<0.0001
**Grade (G) histologic neoplasm**				4963	0.420
G1	5 (1.3)	0 (0.0)	5 (1.0)		
G2	47 (12.2)	18 (19.1)	65 (13.5)		
G3	325 (84.2)	75 (79.8)	400 (83.3)		
G4	1 (0.3)	0 (0.0)	1 (0.2)		
GB	2 (0.5)	0 (0.0)	2 (0.4)		
GX	6 (1.6)	1 (1.1)	7 (1.5)		
**Death**	245	52	297	0.006	0.937
**Time overall survival**	34 (39)	31 (35)	33 (40)	22,653	0.082 ^a^

U: Mann-Whitney test. χ^2^ qui-quadrado. MSI: Microsatellite instability. TMB: tumor mutational burden. MANTIS score: used to predict the MSI value. ^a^ *p* = 0.041, one-tailed Mann-Whitney test.

**Table 2:** Analysis of *Titin* (TTN) gene mutation as predictive to social and clinical variables of women with ovarian cystadenocarcinoma.

Variables	OR	95%CI	*p*	aOR	95%CI	*p*
**Diagnosis Age**	0.994	0.975, 1.014	0.554			
**Race/skin color**			0.251			
Asian	0.208	0.015, 2.854	0.240			
Black or African american	0.152	0.012, 1.940	0.147			
White	0.12	0.011, 1.336	0.085			
**Genetic factors**						
Aneuploidy Score	0.983	0.954, 1.013	0.268			
Buffa Hypoxia Score	1.035	1.012, 1.058	0.003	1.028	0.989, 1.068	0.165
Fraction Genome Altered	1.088	0.348, 3.404	0.885			
MSI MANTIS Score	7.484	0.022, 2.501	0.497			
MSI sensor Score	1.206	1.042, 1.396	0.012	1.089	0.751, 1.580	0.651
Mutation Count	1.010	1.005, 1.014	<0.0001			
Regnum Hypoxia Score	1.029	0.991, 1.068	0.131	0.988	0.934, 1.044	0.658
Winter Hypoxia Score	1.025	1.007, 1.045	0.008	1.007	0.976, 1.039	0.671
TMB nonsynonymous	1.522	1.351, 1.715	<0.0001	1.616	1.346, 1.941	<0.0001
**Grade (G) histologic neoplasm (Reference: GX)**			0.716			
G1	0	0	0.999			
G2	2.298	0.258, 20.442	0.456			
G3	1.385	0.164, 11.673	0.765			
G4	0	0	1.000			
GB	0	0	0.999			
**Dead**	0.982	0.629, 1.533	0.937			
**Time overall survival**	0.993	0.986, 1.001	0.077	0.993	0.982, 1.004	0.211

OR: Odds Ratio. 95%CI: 95% confidence interval. U: Mann-Whitney test. χ^2^ qui-quadrado. MSI: Microsatellite instability. TMB: tumor mutational burden. MANTIS score: used to predict the MSI status.

## Data Availability

All data are available at https://www.cbioportal.org (accessed on 12 October 2022) and https://www.oncokb.org/cancerGenes (accessed on 13 October 2022).
